# Self-reported social media use by adolescents in Brazil: a school-based survey

**DOI:** 10.47626/2237-6089-2022-0545

**Published:** 2024-05-24

**Authors:** Rivka B. Pereira, Thais C. Martini, Claudia Buchweitz, Renata R. Kieling, Helen L. Fisher, Brandon A. Kohrt, Valeria Mondelli, Christian Kieling

**Affiliations:** 1 Departamento de Psiquiatria Hospital de Clínicas de Porto Alegre UFRGS Porto Alegre RS Brazil Serviço de Psiquiatria da Infância e Adolescência, Departamento de Psiquiatria, Hospital de Clínicas de Porto Alegre, Universidade Federal do Rio Grande do Sul (UFRGS), Porto Alegre, RS, Brazil.; 2 Departamento de Pediatria UFRGS Porto Alegre RS Brazil Departamento de Pediatria, UFRGS, Porto Alegre, RS, Brazil.; 3 Institute of Psychiatry, Psychology & Neuroscience Social, Genetic & Developmental Psychiatry Centre King’s College London London UK Institute of Psychiatry, Psychology & Neuroscience, Social, Genetic & Developmental Psychiatry Centre, King’s College London, London, UK.; 4 Economic and Social Research Council Centre for Society and Mental Health King’s College London London UK Economic and Social Research Council, Centre for Society and Mental Health, King’s College London, London, UK.; 5 Division of Global Mental Health Department of Psychiatry and Behavioral Sciences The George Washington University Washington DC USA Division of Global Mental Health, Department of Psychiatry and Behavioral Sciences, The George Washington University, Washington, DC, USA.; 6 Department of Psychological Medicine Institute of Psychiatry, Psychology & Neuroscience King’s College London London UK Department of Psychological Medicine, Institute of Psychiatry, Psychology & Neuroscience, King’s College London, London, UK.; 7 National Institute for Health Research South London and Maudsley NHS Foundation Trust King’s College London London UK Maudsley Biomedical Research Centre, National Institute for Health Research, South London and Maudsley NHS Foundation Trust, King’s College London, London, UK.

**Keywords:** Adolescent, social media, internet use, prevalence

## Abstract

**Objective:**

Although there is a general perception that adolescent social media use is a global phenomenon, there is a scarcity of data on patterns and preferences of social media use among youth in low- and middle-income countries (LMICs). We here describe self-reported prevalence and perceived effects of social media use in a school-based sample of Brazilian adolescents.

**Methods:**

We analyzed cross-sectional data on 7,113 adolescents aged 14 to 16 years enrolled at 101 public state schools between 2018 and 2019 in Porto Alegre, state of Rio Grande do Sul, Brazil.

**Results:**

Of the 7,113 adolescents with complete data for analyses, 54.9% were female, and 60.6% reported their skin color as white. At least one social media platform was used by 97.7% of adolescents every day, and 64.7% reported being online “almost constantly.” YouTube and WhatsApp were the most popular platforms. Most participants perceived the effect on their lives of social media use as neutral.

**Conclusion:**

The pattern of social media use by adolescents in Porto Alegre, Brazil, is similar to that reported for samples from high income countries. Also, we found that those who reported being constantly online were also more likely to report socializing with their friends offline.

## Introduction

The current generation of teenagers and young adults is the first to be raised in highly digitized societies with access to an increasing number of activities on digital devices.^1^ In this scenario, social media has become an important means of communication, entertainment, and leisure for this age group. Nonetheless, there is an ongoing debate about social media’s positive or negative impacts on adolescents’ lives.^2-7^

Although there is a general perception that adolescent social media use is a global phenomenon, remarkably little is known about how adolescents in low and middle-income countries (LMICs) interact with social media platforms,^8^ which could be essential for researchers and practitioners to engage with this population and potentially create effective digital interventions. Therefore, our main objective is to describe the prevalence of self-reported use of social media and the perceived effect of social media use in a school-based sample of adolescents from Brazil, an upper-middle-income country.

## Methods

Students aged 14 to 16 years, enrolled in 101 state public schools in Porto Alegre, state of Rio Grande do Sul, Brazil, were invited to participate in the study. Schools were chosen by convenience, and the questionnaires were administered by two to four researchers in a separate room designated by the school. Adolescents only took part in data collection if their parents/guardians did not disagree to their participation after reading a parent information form (PIF). Further, only adolescents who agreed to participate on the day of administration were included, after providing written informed assent prior to starting the survey. Details on the ascertainment process and data collection procedures are described elsewhere.^9^ Participants answered an eight-item questionnaire regarding frequency of social media use – including Facebook, Instagram, Text/SMS, Twitter, WhatsApp, YouTube, Facebook Messenger, and other social media platforms. Following previous population-based surveys on a similar topic,^10^ the response options were “never,” “once a week or less,” “several times a week,” “once a day,” “several times a day,” and “almost constantly.” Participants subsequently reported their perception of the effect of social media on their lives (“mostly positive,” “neither positive nor negative,” or “mostly negative”), and answered a separate yes/no question on whether they usually meet friends in person to play, chat, or do other things.

Frequency distributions were obtained. Categorical and numerical variables were compared using the chi-square and Mann-Whitney tests, respectively. All analyses were performed in R, version 3.6.1.

### Ethical considerations

Approval for this study was obtained from the Secretaria de Educação do Rio Grande do Sul and from the ethics committee at Hospital de Clínicas de Porto Alegre (HCPA).

## Results

Between July 2018 and December 2019, we assessed 7,720 adolescents, corresponding to about 70% of all students enrolled in the grades eligible for our study at state schools in Porto Alegre at the time of data collection.^11^ Of the 7,113 adolescents with complete data for analyses, 54.9% were female and 60.6% reported their skin color as white. In terms of frequency of social media use, 97.7% of adolescents reported using at least one social media platform every day, and 64.7% reported being online “almost constantly,” with more girls reporting being constantly connected than boys (68.6 vs. 59.8%, respectively; χ^2^(5) = 62.1, p < 0.001).

**Figure 1:**
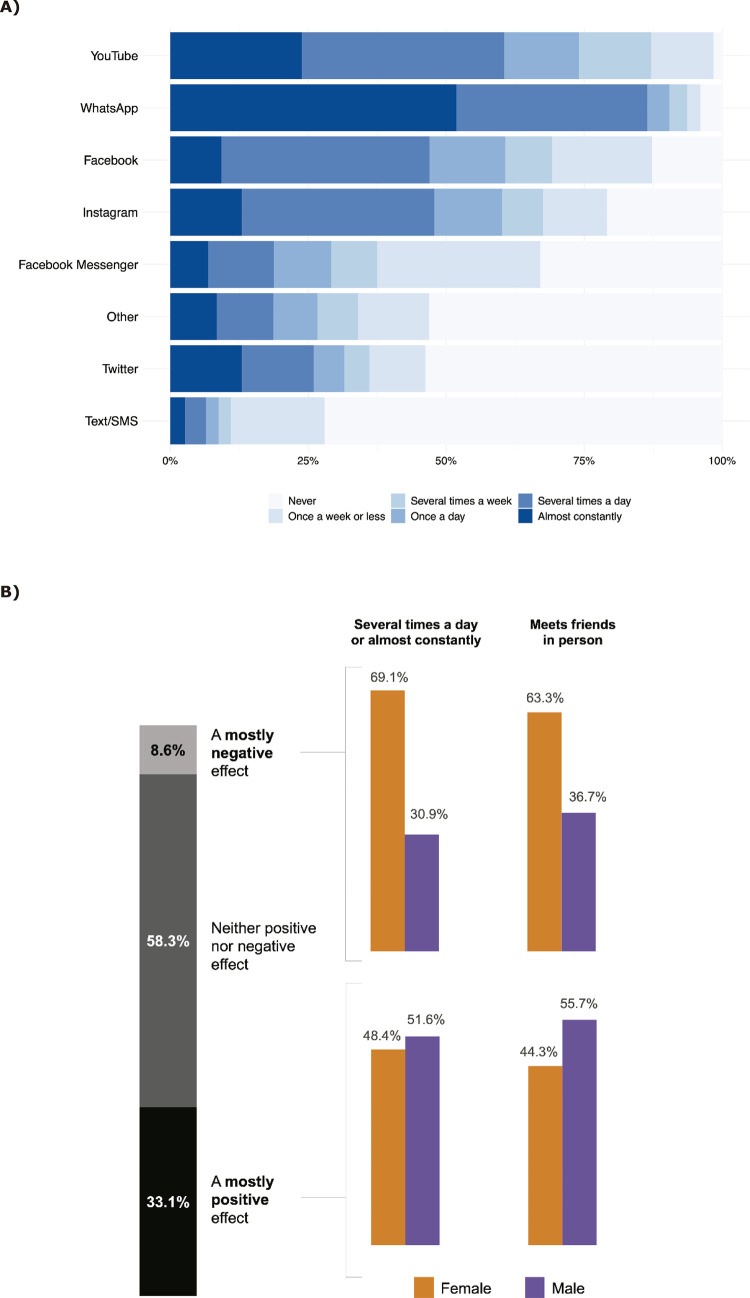
A) Frequency of social media use by platform (n = 7,113). Percentage of social media use by platform across frequency categories. B) Perceived effect of social media use by sex (n = 7,113). “Several times a day or almost constantly” indicates the percentage of participants who reported using social media “several times a day” or “almost constantly” on any of the platforms available in the questionnaire.

Regarding choice of platform, 90.4% of the sample reported using WhatsApp at least once a day, followed by YouTube (74%), Facebook (60.7%), and Instagram (60.1%). Although over 86.5% of participants reported using WhatsApp “several times a day” or “almost constantly,” YouTube was the most cited platform, with 98.4% reporting some use across frequency categories. The least used platforms were Twitter and Text/SMS, with 53.8 and 72% of the sample respectively reporting they “never” use them (Figure 1A).

When asked about the perceived effect of social media on their lives, most adolescents (58.3%) considered the impact as neutral (Figure 1B). Among those who perceived a mostly negative effect (8.6%), girls more often (10.7%) reported feeling negatively impacted by social media than boys (5.9%) (χ^2^[2]=102.8; p < 0.001). Additionally, about 75% of the sample reported usually meeting friends in person, and the frequency of social media use was associated with a higher prevalence of meeting friends in person (U = 375,483; p < 0.001).

## Discussion

Our study identified that the majority of 14-16-year-old adolescents from state schools in the city of Porto Alegre, Brazil, used at least one social media platform every day and that although most of them perceived the effect of social media use on their lives as neutral, one out of nine girls and one out of 17 boys saw it as negative. These findings are in accordance with previous studies showing high use of social media by adolescents worldwide.^12^ The finding of more frequent perceived negative impact of social media in girls than boys might be explained by sex differences in terms of type of online content accessed.^13^ Given the increasing evidence of negative impacts of social media on mental health on some girls in high income countries, such as anxiety, depressive symptoms, and sleep disturbances,^14,15^ our findings suggest that similar detrimental effects may be present in middle-income countries.

Moreover, despite the considerable prevalence of social media use, three out of four adolescents in our sample reported usually meeting friends in person to chat, play games, or do other things. Interestingly, we found that, on average, those who reported being constantly online were also more likely to report socializing with their friends offline. This is in accordance with earlier observations showing that highly connected young people are just as likely as their less-connected peers to regularly interact with friends in person.^10^

These results are not without limitations. For instance, our findings are limited by the self-reported nature of the measures of social media use and the study’s cross-sectional design. Moreover, although this is a large sample, it does not represent all the contexts within the country, which could be explored in future studies, both in Brazil as well as in other LMICs. Also, more recent platforms were not included in the questionnaire (e.g., Snapchat, TikTok). In spite of these limitations, however, our study adds to understanding of social media use by adolescents in an urban area from a middle-income country. Learning which platforms are mostly used by this age group can help us devise effective ways of interacting with them. For example, as the most popular social media platform in this study, WhatsApp might be a useful tool for researchers, clinicians, and policy makers to engage with adolescents, which is an aspect to be explored in future research.
